# Characterization of Nine Compounds Isolated from the Acid Hydrolysate of *Lonicera fulvotomentosa* Hsu et S. C. Cheng and Evaluation of Their In Vitro Activity towards HIV Protease

**DOI:** 10.3390/molecules24244526

**Published:** 2019-12-11

**Authors:** Xia Wang, Ying Wei, Wei-Yi Tian, Meena Kishore Sakharkar, Qing Liu, Xin Yang, Yan-Zi Zhou, Cheng-Li Mou, Gui-Lan Cai, Jian Yang

**Affiliations:** 1 College of Pharmacy, Guizhou University of Traditional Chinese Medicine, South of Dongqing Road, Guiyang 550025, Guizhou Province, China; wangxia1435962672@163.com (X.W.); tianweiyi@gzy.edu.cn (W.-Y.T.); liuqing1994all@163.com (Q.L.); yangxin3153@163.com (X.Y.); mouchengli_h@163.com (C.-L.M.); caiguinan1996@163.com (G.-L.C.); 2 Drug Discovery and Development Research Group, College of Pharmacy and Nutrition, University of Saskatchewan, 107 Wiggins Road, Saskatoon, SK S7N 5E5, Canada; meena.sakharkar@usask.ca; 3 Laboratory of Mesoscopic Chemistry, Institute of Theoretical and Computational Chemistry, Nanjing University, Nanjing 210093, Jiangsu Province, China; zhouyanzi@nju.edu.cn

**Keywords:** *Lonicera fulvotomentosa* Hsu et S. C. Cheng, acid hydrolysate, HIV protease, molecular docking, inhibitor

## Abstract

In this study, we isolated nine compounds from the acid hydrolysate of the flower buds of *Lonicera fulvotomentosa* Hsu et S. C. Cheng and characterized their chemical structures using ^1^H-NMR, ^13^C-NMR, and electron ionization mass spectroscopy (EI-MS). These compounds were identified as β-sitosterol (**1**), 5,5′-dibutoxy-2,2′-bifuran (**2**), nonacosane-10-ol (**3**), ethyl (3β)-3,23-dihydroxyolean-12-en-28-oate (**4**), oleanolic acid (**5**), ethyl caffeate (**6**), caffeic acid (**7**), isovanillin (**8**), and hederagenin (**9**), with **4** as a new triterpene compound. Inhibitory activity against human immunodeficiency virus (HIV) protease was also evaluated for the compounds, and only ethyl caffeate, caffeic acid, and isovanillin (**6**, **7**, and **8**) exhibited inhibitory effects, with IC_50_ values of 1.0 μM, 1.5 μM, and 3.5 μM, respectively. Molecular docking with energy minimization and subsequent molecular dynamic (MD) simulation showed that ethyl caffeate and caffeic acid bound to the active site of HIV protease, while isovanillin drifted out from the active site and dissociated into bulk water during MD simulations, and most of the binding residues of HIV protease have been previously identified for HIV protease inhibitors. These results suggest that caffeic acid derivatives may possess inhibitory activities towards HIV protease other than previously reported inhibitory activities against HIV integrase, and thus ethyl caffeate and caffeic acid could be used as lead compounds in developing potential HIV protease inhibitors, and possibly even dual-function inhibitors against HIV.

## 1. Introduction

*Lonicera fulvotomentosa* Hsu et S. C. Cheng belongs to the Caprifoliaceae (honeysuckle) family. Commonly known as “Shan Yin Hua” or “Ren Dong”, it is widely used as an edible and medicinal food in China [1]. In the versions of Chinese Pharmacopoeia earlier than 2000, *L. japonica* Thunb, *L. macranthoides* Hand.-Mazz., *L. hypoglauca* Miq., *L. confusa* DC. and *L. fulvotnetosa* Hsu et S. C. Cheng were listed in the category designated as *L. Japonicae* Flos (“Jin Yin Hua” in Chinese). Other than being added into liquor and tea in folk diet, the flower buds of *L. Japonicae* Flos are widely used to treat diseases, such as arthritis, colds, enteritis, fever, infections, pains, sores, swelling, and diabetes mellitus [2,3,4,5]. *Lonicera* species have been shown to possess hepatoprotective, antiallergic, anti-inflammatory, antibacterial, and antiviral activities [6,7,8]. Various active ingredients, such as caffeoylquinic acid, secoiridoids, flavonoids, cerebrosides, nitrogen-containing iridoid glycosides, and triterpene glycosides, have been isolated and characterized from *Lonicera* species [9,10,11,12,13,14]. In addition, a number of triterpene derivatives, caffeoylquinic acid derivatives, and flavonoids have exhibited potent inhibitory effects against human immunodeficiency virus (HIV)-1 integrase and prevented HIV-1 replication in tissue culture [15,16,17,18,19]. However, in vitro binding and inhibition of HIV-1 integrase does not always translate into potency against HIV replication [20,21]. In this study, we isolated and characterized nine compounds from the acid hydrolysate of the flower buds of *L. fulvotomentosa* Hsu et S. C. Cheng and evaluated their respective anti-HIV-protease activity under in vitro conditions, aiming to verify whether inhibition of HIV protease is also involved in the anti-HIV function of *Lonicera* species. Out of the nine compounds, ethyl caffeate and caffeic acid were identified to possess the highest inhibitory effects against HIV protease with respective IC_50_ values of 1.0 μM and 1.5 μM, suggesting that they could be used as lead compounds to develop more potent anti-HIV protease agents.

## 2. Results and Discussion

### 2.1. Characterization of the Compounds

**Figure 1:**
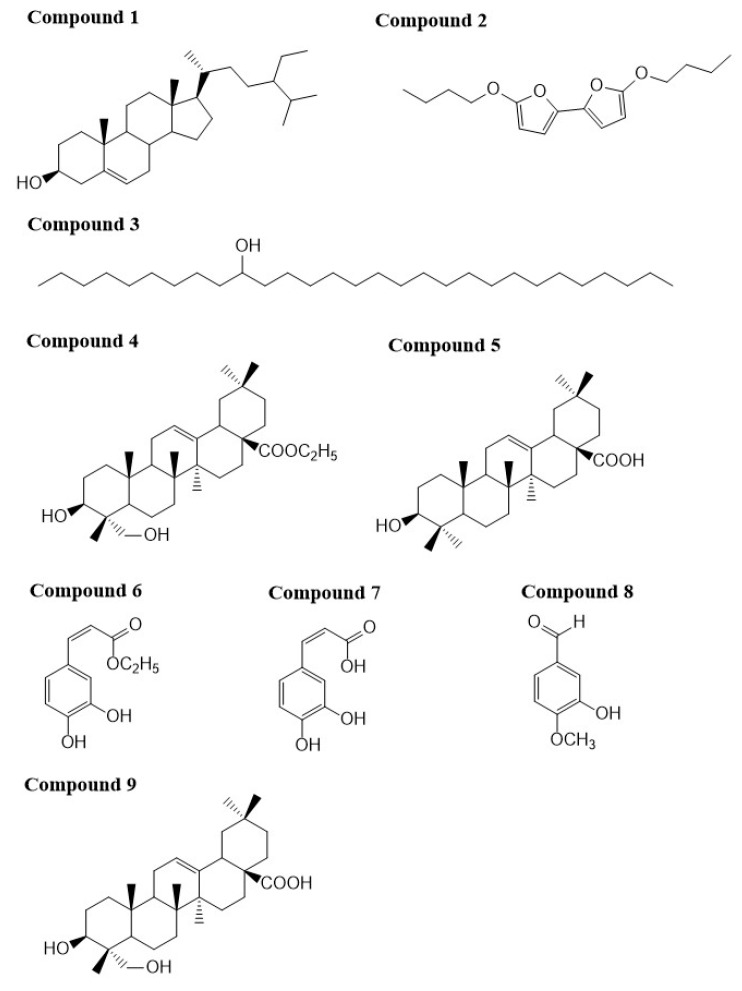
Chemical structures of the nine compounds isolated from the acid hydrolysate of the flower buds of *L. fulvotomentosa* Hsu et S. C. Cheng.

**Figure 2:**
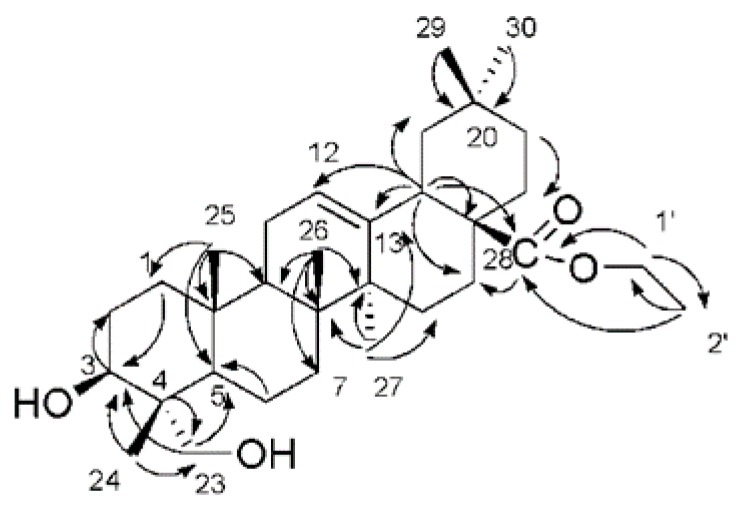
Key HMBC spectrum of compound **4**.

*Lonicera* species possess antiviral activities [22]. Triterpene derivatives, caffeoylquinic acid derivatives, and flavonoids isolated from *Lonicera* species exhibit potent inhibitory effects against human immunodeficiency virus (HIV)-1 integrase and prevented HIV-1 replication in tissue culture [15,16,17,18,19]. However, there is also evidence showing that strong binding to HIV-1 integrase does not always translate into potent antiviral activity [20,21]. To investigate whether *L. fulvotomentosa* Hsu et S. C. Cheng contains any molecules that can be used as lead compounds in developing potent HIV-protease inhibitors, we isolated nine compounds from the acid hydrolysate of its dried flower buds. The compounds were subsequently characterized using ^1^H-NMR and ^13^C-NMR spectroscopy and electron ionization mass spectroscopy (EI-MS). As shown in Figure 1, the nine compounds were identified as β-sitosterol (**1**), 5,5′-dibutoxy-2,2′-bifuran (**2**), nonacosane-10-ol (**3**), ethyl (3β)-3,23-dihydroxyolean-12-en-28-oate (**4**), oleanolic acid (**5**), ethyl caffeate (**6**), caffeic acid (**7**), isovanillin (**8**), and hederagenin (**9**). Only compound **4** is a new triterpene molecule. Chemical structures of the other compounds have already been reported [23,24,25,26,27,28,29,30], and therefore were not discussed again in this report. Alongside ^1^H-NMR, ^13^C-NMR, and EI-MS, compound **4** was further characterized using heteronuclear multiple quantum coherence (HMQC), heteronuclear multiple bond coherence (HMBC), gradient correlation spectroscopy (GCOSY), and high resolution mass spectroscopy (HR-MS), as seen in Supplementary Figure S1. The chemical structural parameters of compound **4** are as follows: white amorphous powder, [α]_D_^25.9^ = +60° (*C* = 0.05, CHCl_3_); UV *λ*_max_ nm (log *ε*): 242 (3.2); IR *υ*_max_^KBr^ cm^−1^: 3425.8 (C–OH), 2927 (C–H), 1725.17 (C=O); ^1^H and ^13^C-NMR data see Table 1; and HREIMS *m/z* 523.3757 [M+Na]^+^ (calculated for C_32_H_52_O_4_Na, 523.3756). The ^1^H- and ^13^C-NMR spectra were closely resemble those of hederagenin except for the signals appeared at *δ*H 4.10 (H-1′), 1.23 (H-2′) and at *δ*C 60.2 (C-1′), 14.4 (C-2′), implicating the presence of an acetyl group in compound **4** [31]. Long-range correlations were identified between one acetyl proton signal at *δ*H 4.10 (H-1′) and carbon signals at *δ*C 177.5 (C-28) and 14.4 (C-2′); between proton signal at *δ*H 2.86 (H-18) and carbon signals at *δ*C 122.5 (C-12), 143.5 (C-13), 46.4 (C-17) and 177.5 (C-28); and between proton signal at *δ*H 1.23 (H-2′) and carbon signal at *δ*C 60.2 (C-1′), respectively, as seen in Figure 2. These evidences indicate that an acetyl group is linked at C-28. Based on the above analyses, compound **4** was determined to be ethyl (3β)-3,23-dihydroxyolean-12-en-28-oate.

### 2.2. Inhibitory Activity against HIV Protease

To evaluate whether any of the nine compounds could potentially be used as a lead compound in developing potent HIV-protease inhibitors, their inhibitory effect against HIV protease was evaluated with the SensoLyte™ 520 HIV PR Assay Kit Fluorimetric using previously published protocols [32]. As shown in Table 2, only ethyl caffeate (**6**), caffeic acid (**7**), and isovanillin (**8**) showed inhibitory activities against HIV protease. At a concentration of 1.0 mg/mL, compounds **6**, **7**, and **8** exhibited 100 ± 12.8%, 90.2 ± 7.3%, and 61.2 ± 2.8% inhibition of HIV protease, respectively. Their respective IC_50_ values were measured to be 1.0 μM, 1.5 μM, and 3.5 μM. Compared to the positive control (pepstatin A, IC_50_ = 0. 016 μM), compounds **6**, **7**, and **8** were weaker inhibitors of the HIV protease. To our knowledge, this is the first report to show that caffeic acid and its derivatives possess inhibitory activities towards HIV protease, although previous studies have shown that they exhibit inhibitory effects against HIV integrase [33]. The low micromolar IC_50_ values for ethyl caffeate, caffeic acid, and isovanillin warrant further investigations to develop more potent caffeic acid-based inhibitors against HIV protease. Furthermore, it would be even more advisable to develop caffeic acid-based dual-function inhibitors, which can inhibit both HIV protease and HIV integrase. The current study is consistent with the observation that coffee has a protective effect on mortality of HIV/AIDS patients [34].

### 2.3. Molecular Docking and Molecular Dynamic Simulation

**Figure 3:**
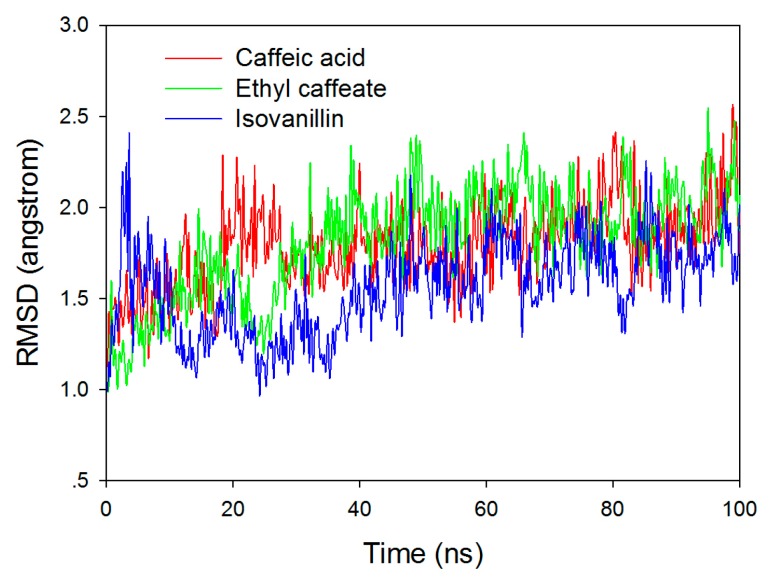
Root mean square deviation (RMSD) of backbone heavy atoms of HIV relative to the crystal structure in complex with ethyl caffeate, caffeic acid, and isovanillin, respectively, from 100 ns classical MD simulations.

To gain insight into how caffeic acid and its derivatives inhibit HIV protease, we docked ethyl caffeate (**6**), caffeic acid (**7**), and isovanillin (**8**) to the active site of HIV protease (PDB ID: 1QBS) [35] with a grid box of 20 × 20 × 20 Å, using pepstatin A as a positive control. Both Schrödinger (Cambridge, MA, USA) and SYBYL-x (Certara Inc., Princeton, NJ, USA) were used to cross-validate the docking results. Almost identical docking results were obtained from both software. The top five docking poses for each molecule from Schrödinger, as seen in Supplementary Table S1, were subsequently subjected to molecular dynamic (MD) simulations. To verify the stability of the HIV protease–inhibitor complexes, we conducted 100 ns classical MD simulations for ethyl caffeate, caffeic acid, and isovanillin binding to HIV protease. The root mean square deviation (RMSD) values of backbone heavy atoms of HIV protease are shown in Figure 3 with respect to the crystal structure. After several ns of simulation, the HIV protease reached equilibration.

**Figure 4:**
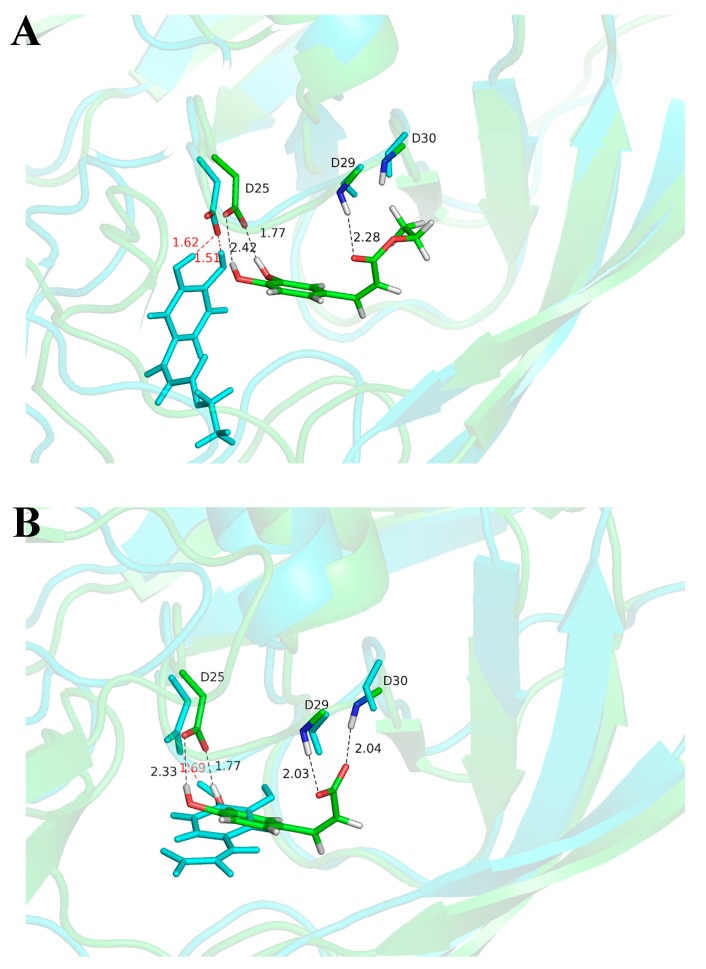
Overlap of the structures of docked molecules and the snapshots after 100 ns MD simulations for HIV protease in complexed with (**A**) ethyl caffeate and (**B**) caffeic acid, respectively. The carbon atoms are colored green for the docked structures, and cyan for the MD snapshots. The distances of hydrogen bonds are labeled in black for docked structures and red for MD snapshots, respectively.

**Figure 5:**
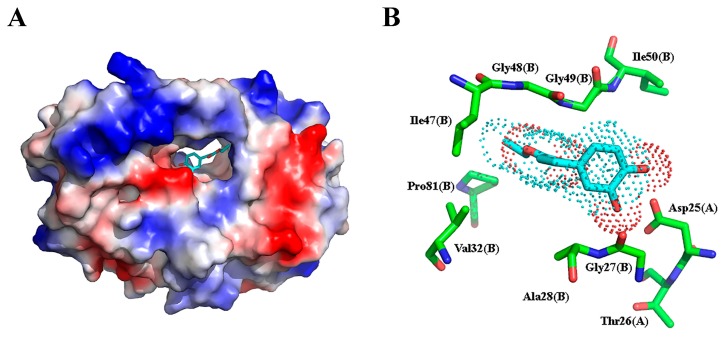
Electrostatic potential of HIV protease with ethyl caffeate (shown in stick model and after 100 ns MD simulation) located in the solvent channel formed by the two HIV protease monomers, with blue and red representing positive and negative electrostatic potentials, respectively (**A**), and HIV protease amino acid residues involved in the interaction with ethyl caffeate (**B**).

Isovanillin drifted out from the binding pocket and dissociated into bulk water after only a few ns of simulation, while the other two inhibitors stayed within the binding pocket after 100 ns MD simulations. In order to obtain conformational variations for ethyl caffeate and caffeic acid, we compared the initial docking modes with the snapshots after 100 ns MD simulations, as seen in Figure 4. After molecular docking, the hydroxyl groups of either ethyl caffeate or caffeic acid formed two hydrogen bonds with the carboxyl group of Asp25(A) (i.e., Asp25 from HIV protease monomer A). The two oxygen atoms of the carboxyl group of caffeic acid formed hydrogen bonds with the NH groups of residues Asp29(A) and Asp30(A), respectively, while the carbonyl oxygen of ethyl caffeate formed one hydrogen bond with Asp29(A). However, after 100 ns MD simulations, conformations of the two inhibitors changed significantly although they were still located within the binding pocket of HIV protease. It is clearly visible that the carboxyl group of caffeic acid was repelled by the negative charged side chains of Asp29(A) and Asp30(A) and there was only one hydrogen bond remained between one hydroxyl group of caffeic acid and Asp25(A), as seen in Figure 4B. For ethyl caffeate, the two hydrogen bonds remained with shorter H-bond distances between its two hydroxyl groups and Asp25(A), as seen in Figure 4A. After 100 ns MD simulation, ethyl caffeate was still bound to the active site of HIV protease, which is located in the solvent channel with negative electrostatic potentials formed by the two HIV protease monomers, as seen in Figure 5A. The interaction between ethyl caffeate and HIV protease is predominated by amino acid residues from monomer B, as seen in Figure 5B. The amino acid residues involved in the ethyl caffeate binding include residues Asp25 and Thr26 from monomer A and residues Gly27, Ala28, Val32, Ile47, Gly48, Gly49, Ile50, and Pro81 from monomer B. Most of these amino acid residues have been previously identified to be involved in binding HIV protease inhibitors, such as indinavir, and be responsible for drug resistance upon mutations [36]. Crystallographic studies of ethyl caffeate, as well its derivatives, bound to HIV protease and integrase and in vitro and in vivo studies of the inhibitory effects of caffeic acid derivatives on HIV viral replication would be extremely beneficial in confirming these interactions and guiding the development of more potent caffeic acid-based HIV protease inhibitors and even possibly dual-function inhibitors.

## 3. Materials and Methods 

### 3.1. Materials

Dried flower buds of *L. fulvotomentosa* Hsu et S. C. Cheng were purchased from Xinyi Medicine Market (Guiyang, Guizhou Province, P. R. of China) in 2016, and a voucher sample (No. 0716/LF) was deposited in the College of Pharmacy, Guizhou University of Traditional Chinese Medicine (Guiyang, Guizhou Province, P. R. of China). SensoLyteTM 520 HIV PR Assay Kit Fluorimetric (Lot#: AK 71147-1021) and HIV protease (Lot#: 129-067) were purchased from AnaSpec Inc. (San Jose, CA, USA). Corning Microtest 384-well 120 μL black assay plates (Lot#: 09818015) were purchased from Corning Inc. (Corning, NY, USA). Silica gel (100–200 mesh) was purchased from Qingdao Marine Chemical Inc. (Qingdao, Shandong Province, P. R. of China) and octadecylsilane (ODS, 100–200 mesh, Lot#: R060311) was purchased from Fuji Silysia Chemical Ltd. (Aichi, Japan). All chemical reagents were purchased from Aladdin Chemistry Co. Ltd. (Shanghai, P. R. of China).

### 3.2. Extraction and Isolation

Dried flower buds of *L. fulvotomentosa* Hsu et S. C. Cheng (20.0 kg) were refluxed twice using MeOH–H_2_O (7:3) for 1.5 h. The MeOH extracts were combined, concentrated under reduced pressure, and refluxed using 2.0 M H_2_SO_4_ in aq. EtOH (1:1) for 3 h. The solution was neutralized to pH 7.0 using 2.0 M NaOH and extracted thrice using an equal volume of CHCl_3_. The CHCl_3_ organic layers were combined and evaporated to obtain a dark and oleaginous material (1528.1 g). Subsequently, the CHCl_3_ extract was subjected to silica gel column chromatography (114 cm × 7.8 cm) to yield 308 fractions using a hexane-ethyl acetate gradient elution (100:0 → 50:1 → 0:100).

Fraction2 (1.5 g) was subjected to further purification using a silica gel column (64 cm × 3.0 cm) with a hexane–ethyl acetate gradient elution (100:0 → 20:1). Compound **1** (987 mg) was obtained from recrystallization with ethyl acetate. Fraction11 (1.8 g) was also subjected to silica gel column chromatography (64 cm × 3.0 cm) to obtain Fraction11A22-25 (195 mg) with a hexane–ethyl acetate gradient elution (100:0 → 50:1). Fraction11A22-25 was then purified using a preparative thin-layer chromatography (hexane–ethyl acetate = 80:1) to obtain compound **2** (65 mg). Fraction14 (850 mg) was applied to a silica gel column (75 cm × 3.5 cm) and eluted with a hexane–ethyl acetate gradient (100:0 → 40:1) to obtain 38 fractions, and Fraction14A13 was further recrystallized to obtain compound **3** (18 mg). Fraction38 (10.5 g) was subjected a silica gel column chromatography (75 cm × 3.5 cm) with a hexane–ethyl acetate gradient elution (100:0 → 30:1) to obtain compound **4** (6.3 g). Fraction56 (2.324 g) was purified first by a silica gel column (50 cm × 3.0 cm) with a CH_2_Cl_2_–MeOH gradient elution (100:0 → 80:1) and then by a Sephadex LH-20 column (150 cm × 2.5 cm) to obtain compound **5** (25 mg). Fraction65-69 (15.0 g) was also subjected to further purification using silica gel column chromatography (75 cm × 3.5 cm) with a CH_2_Cl_2_–MeOH gradient elution (100:0 → 40:1), and compound **6** (4.481 g) and compound **7** (1.36 g) were obtained from Fraction65-69B15-52 and Fraction65-69D8-22, respectively. Fraction85 (1.2 g) was purified first by ODS column chromatography (40 cm × 1.5 cm) with a MeOH gradient elution (50% → 100%) and further by silica gel column chromatography (30 cm × 2.0 cm) with a CH_2_Cl_2_–MeOH gradient elution (40:1 → 0:100) to obtain compound **8** (15 mg). Fraction124-265 (279 g) was subjected to silica gel column chromatography (100 cm × 4.0 cm) with a CH_2_Cl_2_–MeOH gradient elution (50:1 → 15:1) to obtain compound **9** (24.5 g).

### 3.3. Compound Characterization

The melting point of the nine purified compounds was determined using an X-4A melting point microscopic apparatus (Kerui Instrument Company, Zhengzhou, Henan Province, P. R. of China). Optical rotation of the compounds was measured using an automatic polarimeter (Rudolph Research Analytical, Hackettstown, NJ, USA). The purified compounds were characterized by ^1^H-NMR, ^13^C-NMR, and electron ionization mass spectroscopy (EI-MS) using published protocols [32]. Briefly, ^1^H- and ^13^C-NMR were measured using a JEOL JHA-LAA 400 (^1^H, 400 MHz; ^13^C, 100 MHz) spectrometer (JEOL USA Inc., Peabody, MA, USA) and a Bruker DRX 500 (^1^H, 500 MHz; ^13^C, 125 MHz) spectrometer (Bruker BioSpin Corporation, Fällanden, Switzerland). The chemical shifts were represented as ppm with tetramethylsilane as the internal standard. EI-MS was performed on a VG Auto Spec-3000 spectrometer (Agilent Technologies Comp., Palo Alto, CA, USA). For compound **4**, which is a new triterpene compound, was further characterized with heteronuclear multiple quantum coherence (HMQC), heteronuclear multiple bond coherence (HMBC), gradient correlation spectroscopy (GCOSY), and high-resolution mass spectrometry (HR-MS), as seen in Supplementary Figure S1.

### 3.4. Anti-HIV Protease In Vitro Assay

Inhibitory activity of the purified compounds against HIV protease was assayed with the SensoLyteTM 520 HIV PR Assay Kit Fluorimetric using previously published protocols [32]. Briefly, 2 μL compound solution (in DMSO) and 8 μL freshly diluted HIV protease (0.01 μg/μL) were added to each well of a 384-well black microplate. The proteolytic reaction was initiated by adding 10 μL freshly diluted substrate (100× dilution of a DMSO stock). After incubation at 37 °C for 30 min, fluorescence were measured at Ex/Em = 485 nm/528 nm using a Synergy II Multi-Mode Microplate Reader (BioTek^®^ Instruments Inc., Winooski, VT, USA). Pepstatin A, a known HIV protease inhibitor, was used as a positive control.

### 3.5. Molecular Docking

Molecular docking of compounds **6**, **7**, and **8** to HIV protease was performed with both Schrödinger (Cambridge, MA, USA) and SYBYL-x 2.1.1 (Certara Inc., Princeton, NJ, USA). The crystal structure of HIV protease (PDB ID: 1QBS) was selected as the target for the docking studies [35]. All water molecules and hetero molecules were removed and a grid box of 20 Å × 20 Å × 20 Å was generated around the active site of the HIV protease. Pepstatin A, an FDA-approved HIV protease inhibitor, was used as a positive docking control. All conformers of the three compounds and pepstatin A were included in the rigid receptor docking with OPLS2005 (GLIDE HTVS) force field by Schrödinger and AMBER7 FF99 field (energy minimization) by SYBYL-x. The top five docking conformations for each molecule were reported in the current study, as seen in Supplementary Table S1.

### 3.6. Initial Structure Preparation and Molecular Dynamic Simulation

The initial binding conformations used for MD simulation were obtained from the best docking structures for ethyl caffeate, caffeic acid and isovanillin by Schrödinger. The protonation state of charged residues in the HIV protease was determined at constant pH 7.0 based on *pK_a_* calculations via the PROPKA program [37] and consideration of the local hydrogen bonding network. His69 was identified as HID (i.e., hydrogen on the delta nitrogen). Asp and Glu residues were deprotonated, while Lys and Arg were protonated. The partial charges of the inhibitors were fitted with HF/6-31G(d) [38] calculations and the restrained electrostatic potential (RESP) protocol [39] was implemented using the Antechamber module in Amber16 package. The force field parameters for the inhibitors were adapted from the standard general amber force field (GAFF) parameters. The standard amber99SB [40,41] force field was employed for the protein. Then, the HIV-inhibitor complexes were solvated into a rectangular TIP3P water box with a 12 Å buffer distance on each side and neutralized by additional Cl^−^ ions. Each system was equilibrated with a series of minimizations interspersed by short molecular dynamics simulations, during which restraints on the inhibitors and protein backbone heavy atoms were gradually released. Finally, an extensive molecular dynamics simulation of 100 ns at constant temperature and pressure was carried out. The pressure was maintained at 1 atm and coupled with isotropic position scaling. The temperature was controlled at 310 K with the Berendsen thermostat method [42]. Long-range electrostatic interactions were treated with particle mash Ewald (PME) method [43,44] and 12 Å cutoff was used for both PME and van deer Waals (vdW) interactions. A time step of 1 fs was employed, and a periodic boundary condition was used. All MD simulations were performed using Amber16 molecular dynamic package [45]. 

### 3.7. Statistical Analysis

All experiments in the current study were carried out in triplicate. IC_50_ values of the active compounds were calculated from the plot of averaged percentage of inhibition versus compound concentration. The percentage of inhibition was calculated as *Inhibition* (%) = (*F_vehicle_* − *F_sample_*)/*F_vehicle_* × 100, where *F_vehicle_* and *F_sample_* are the fluorescence values of vehicle control minus the substrate control and of sample minus the substrate control, respectively. All data are reported as mean ± SD. 

## 4. Conclusions

In this study, we isolated and characterized nine compounds from the acid hydrolysate of the flower buds of *L. fulvotomentosa* Hsu et S. C. Cheng, and evaluated their respective in vitro inhibitory activity against HIV protease. Compound **4**, ethyl (3β)-3,23-dihydroxyolean-12-en-28-oate, was identified as a new triterpene compound. Furthermore, ethyl caffeate (**6**), caffeic acid (**7**), and isovanillin (**8**) exhibited potent inhibitory effects towards HIV proteases with IC_50_ values of 1.0 μM, 1.5 μM, and 3.5 μM, respectively. Molecular docking and molecular dynamic simulation showed that ethyl caffeate and caffeic acid bound to the active site of HIV protease and the binding residues of HIV protease were confirmed from previous studies on HIV protease inhibitors. To our knowledge, this is the first report showing that caffeic acid and its derivatives possess inhibitory effects towards HIV protease other than the previously reported inhibitory activities against HIV-1 integrase.

## Figures and Tables

**Table 1:** ^1^H-NMR and ^13^C-NMR spectroscopic data for compound **4**.

Position	*δ*_H_	*δ*_C_	Position	*δ*_H_	*δ*_C_
1	0.90 (m) 1.44 (m) ^a^	38.1	17		46.4
2	1.16 (m) ^b^ 1.98 (m) ^c^	26.6	18	2.86 (d, 8.0)	41.7
3	3.63 (dd, 8.0, 16.0)	76.8	19	a, 1.12 (m) ^d^ b, 1.67 (m)	46.1
4		41.2	20		30.6
5	1.13 (m) ^d^	49.7	21	a, 1.08 (m) b, 1.33 (m) ^e^	33.1
6	a, 1.30 (m) ^e^ b, 1.40 (m)	18.3	22	a, 1.73 (m) b, 1.84 (m)	32.4
7	a, 1.26 (m) b, 1.44 (m) ^a^	32.4	23	a, 3.42 (d, 8.0) b, 3.72 (d, 8.0)	72.1
8		39.3	24	0.89 (s)	11.0
9	1.55 (m)	47.7	25	0.92 (s)	15.8
10		36.9	26	0.74 (s)	16.9
11	a, 1.35 (m) b, 1.95 (m) ^c^	25.8	27	1.13 (s) ^d^	25.8
12	5.28 (t, 3.5)	122.5	28		177.5
13		143.5	29	1.26 (s)	29.6
14		41.7	30	1.26 (s)	29.6
15	a, 1.16 (m) ^b^ b, 1.66 (m)	27.6	1’	4.08 (t, 8.0,16.0)	60.2
16	a, 1.96 (m) b, 2.04 (m)	23.5	2’	1.23 (s)	14.2

^a–e^ Signals bearing the same superscript were overlapped.

**Table 2:** Inhibitory activity of isolated compounds (mean ± RSD) against human immunodeficiency virus (HIV)-1 protease (*n* = 3).

Compound No.	Compound Name	1.0 mg/mL	0.1 mg/mL	0.01 mg/mL	IC_50_ (μM)
**1**	β-Sitosterol	-	-	-	-
**2**	5,5′-Dibutoxy-2,2′-bifuran	-	-	-	-
**3**	Nonacosane-10-ol	-	-	-	-
**4**	Ethyl (3β)-3,23-dihydroxyolean-12-en-28-oate	-	-	-	-
**5**	Oleanonic acid	-	-	-	-
**6**	Ethyl caffeate	100 ± 12.8%	26.8 ± 1.6%	-	1.0
**7**	Caffeic acid	90.2 ± 7.3%	17.3 ± 5.7%	-	1.5
**8**	Isovanillin	61.2 ± 2.8%	20.1 ± 4.6%	-	3.5
**9**	Hederagenin	-	-	-	-
Positive control	Pepstatin A	53.2 ± 3.6% (0.02 μM)	39.2 ± 1.8% (0.002 μM)	29.1 ± 12.5% (0.0002 μM)	0.016

## Supplementary Materials

The following are available online, Table S1: Docking score, GLIDE score and GLIDE Emodel of ethyl caffeate, caffeic acid and isovanillin isolated from *Lonicera fulvotomentosa* Hsu et S. C. Cheng towards HIV protease; Figure S1: Characterization of compound **4**.
