# Supplementary material for: Characterization of Nine Compounds Isolated from the Acid Hydrolysate of Lonicera fulvotomentosa Hsu et S. C. Cheng and Evaluation of Their In Vitro Activity towards HIV Protease
Source: Molecules. 2019 Dec 11;24(24):4526. doi: 10.3390/molecules24244526 (PMC6943440; doi:10.3390/molecules24244526)
Supplement: Supplementary file 1 [file molecules-24-04526-s001.pdf]

Supplementary materials

Table S1. Docking score, GLIDE score and GLIDE Emodel of ethyl caffeate, caffeic acid and isovanillin isolated from *Lonicera fulvotomentosa* Hsu et S. C. Cheng towards HIV protease.

	**HIV protease**		
Compounds	Docking score	GLIDE score	GLIDE Emodel (Kal/mol)
**Ethyl caffeate**			
Pose 1	-5.841	-5.844	-44.875
Pose 2	-5.573	-5.576	-44.431
Pose 3	-5.470	-5.473	-44.139
Pose 4	-5.311	-5.314	-44.925
Pose 5	-5.248	-5.251	-46.826
**Caffeic acid**			
Pose 1	-5.576	-5.576	-39.332
Pose 2	-5.505	-5.505	-41.301
Pose 3	-5.447	-5.447	-39.747
Pose 4	-5.222	-5.222	-37.197
Pose 5	-5.090	-5.090	-36.156
**Isovanillin**			
Pose 1	-6.109	-6.112	-34.995
Pose 2	-6.015	-6.019	-33.160
Pose 3	-5.986	-5.989	-34.585
Pose 4	-5.537	-5.540	-34.236
Pose 5	-5.507	-5.540	-31.349

Supplementary materials

Figure S1. Characterization of Compound 4

^{1}H-NMR

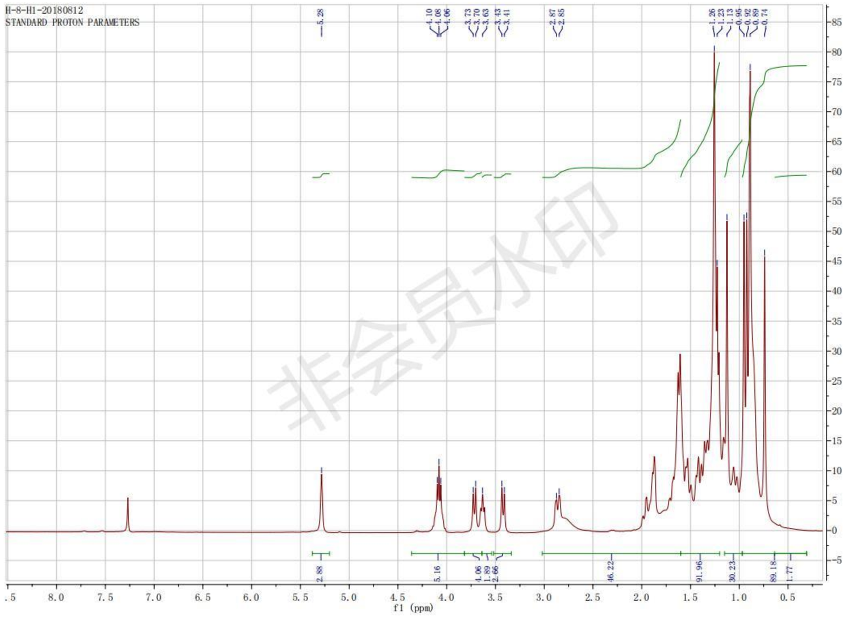

^{13}C-NMR

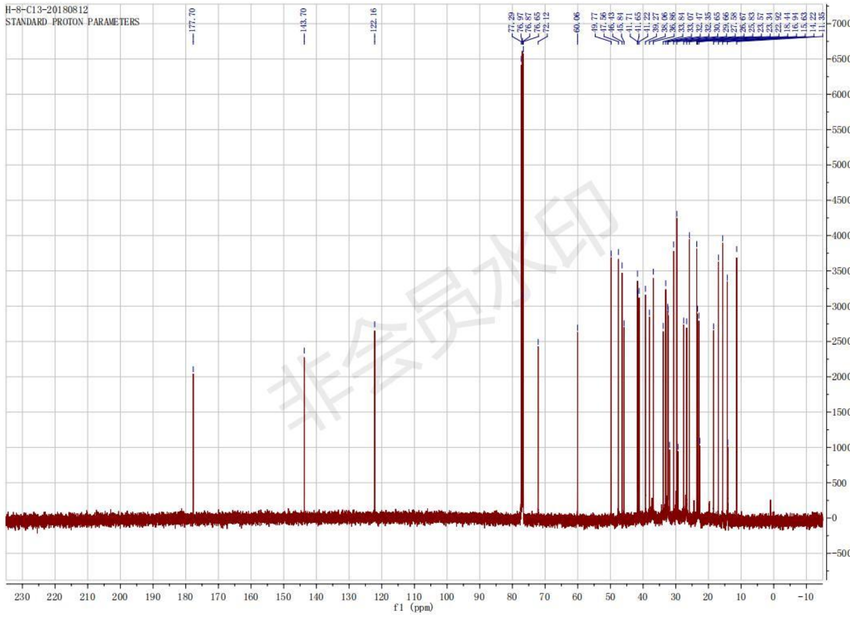

HMQC

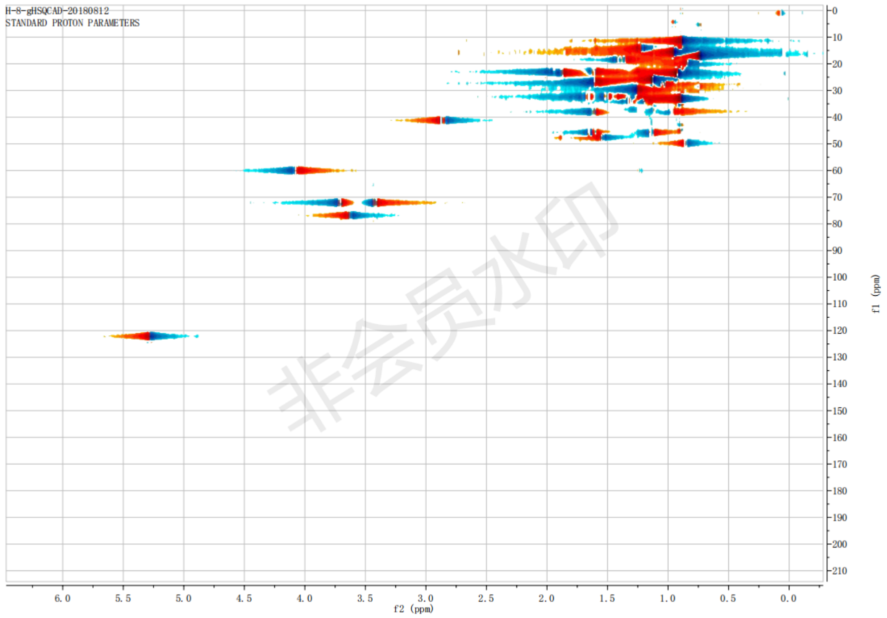
H-8-gHSQCAD-20180812
STANDARD PROTON PARAMETERS
f2 (ppm)
f1 (ppm)

HMBC

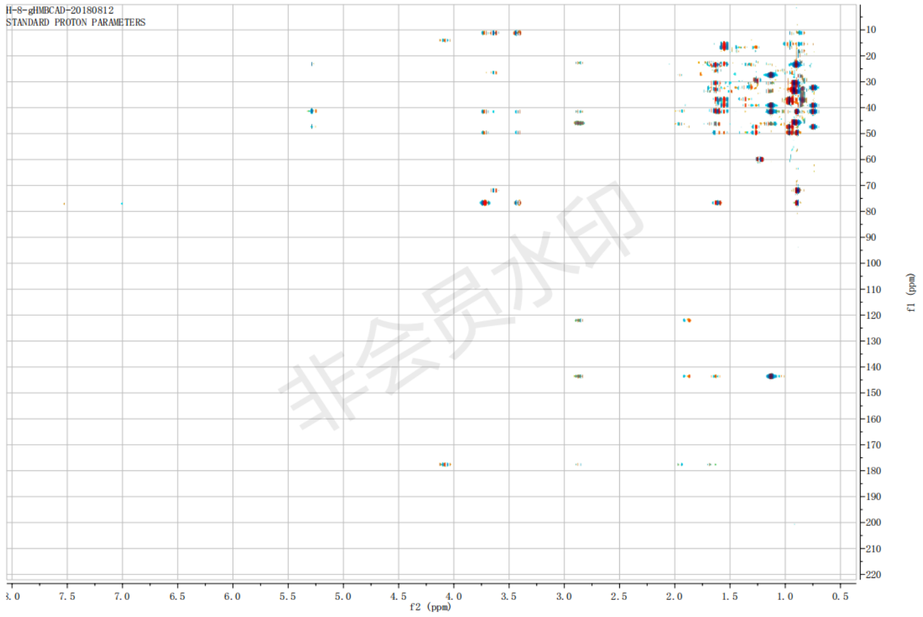
H-8-gHMBCAD-20180812
STANDARD PROTON PARAMETERS
f2 (ppm)
f1 (ppm)

GCOSY

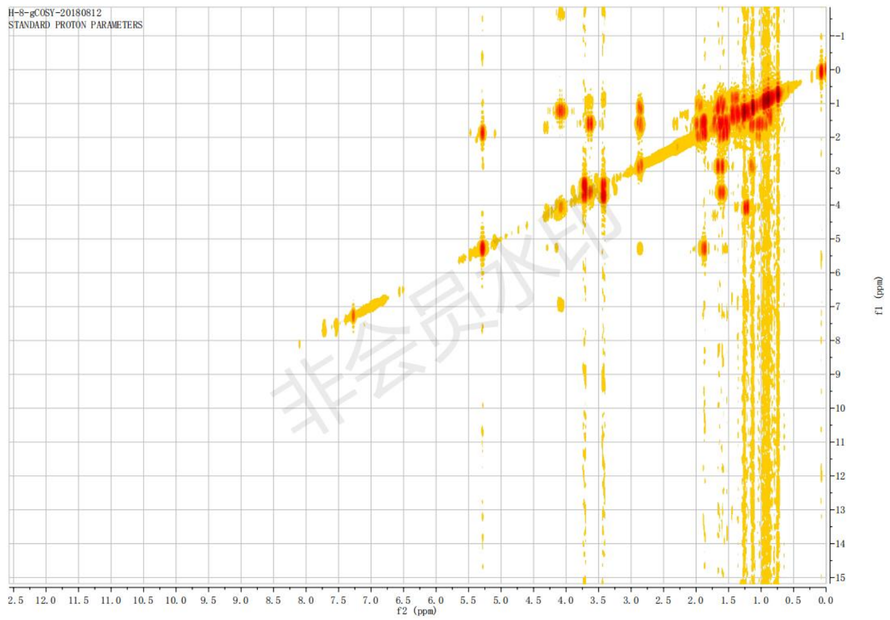

HR-MS

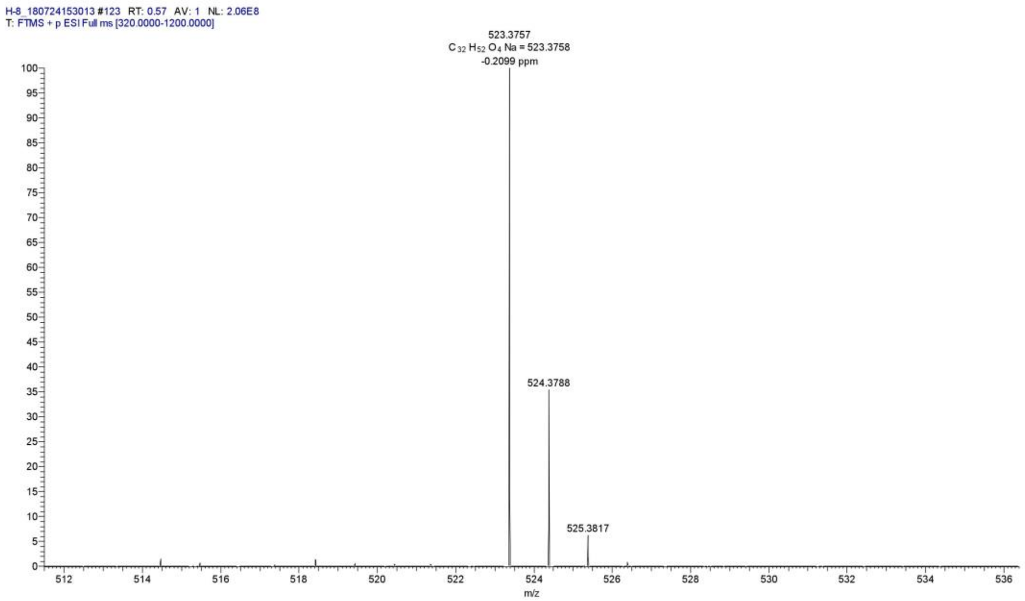

H-8_180724153013 #123 RT: 0.57 AV: 1 NL: 2.06E8
T: FTMS + p ESI Full ms [320.0000-1200.0000]
523.3757
C 32 H 52 O 4 Na = 523.3758
-0.2099 ppm
524.3788
525.3817
m/z
